# Supplementary material for: Relationships among COVID-19 causal factors perceived by children, basic psychological needs and social anxiety
Source: PeerJ. 2025 Jan 31;13:e18828. doi: 10.7717/peerj.18828 (PMC11789665; doi:10.7717/peerj.18828)
Supplement: Supplemental Information 3 [file peerj-13-18828-s003.docx]

**Codebook**

Cell A: Register

Identification number

Cell B: Gender

0,00= Female

1,00= Male

Cell C: Curse

1,00= First cycle of elementary school

2,00= Second cycle of elementary school

3,00= Third cycle of elementary school

Cell D: Borg scale (with mask)

0= Total rest

1= Very light effort

2= Gentle effort

3= Moderate effort

4= A little hard effort

5-6= Hard effort

7-8-9= Very hard effort

10= Max effort

Cell E: Borg scale (without mask)

0= Total rest

1= Very light effort

2= Gentle effort

3= Moderate effort

4= A little hard effort

5-6= Hard effort

7-8-9= Very hard effort

10= Max effort

Cell F: VO2MAX (with mask)

Cell G: VO2MAX (without mask)

Cell H: Period (with mask)

Cell I: Period (without mask)

CELL J: Basal Heart Rate (with mask)

Cell K: Basal Heart Rate (without mask)

Cell L: Heart rate at the end of the test (with mask)

Cell M: Heart rate at the end of the test (without mask)

Cell N:Heart rate one minute after finishing the test (with mask)

Cell O:Heart rate one minute after finishing the test (without mask)

Cell P:Heart rate five minutes after finishing the test (with mask)

Cell Q:Heart rate five minutes after finishing the test (without mask)

Cell R-BW: Poms Scale (without mask). R: Tense; S: Angry; T: Tired; U: Unhappy; V:Animated; W: Confused; X: Hurt by past actions; Y: Agitated; Z: Apathetic; AA: Mad; AB: Sad; AC: Active; AD: To burst; AE: Irritable; AF: Dejected; AG:Energetic; AH: Out of control; AI: Hopeless; AJ: Relaxed; AK: Clumsy; AL: Rancorous; AM: Uneasy; AN:Restless; AO: Lonely; AP: Unlucky; AQ: Stunned; AR: Cheerful; AS: Embittered; AT: Exhausted; AU: Anxious; AV: Fighter; AW: Depressed; AX: Desperate; AY: Thick; AZ: Rebel; BA: Helpless; BB: Without strength; BC: Disorientated; BD: Alert ;BE: Upset; BF: Furious; BG: Efficient; BH: Full of energy; BI: Bad tempered; BJ: Useless; BK: Forgetful; BL: Unable to concentrate; BM: Tired; BN: Annoying; BO: Discouraged; BP: Resentful; BQ: Nervous; BR: Carefree; BS: Terrified; BT:Guilty; BU: Vigorous; BV: Insecure; BW: Weary

0= nothing

1= Little

2= moderately

3= quite

4= very much

Cell BX-EC: Poms Scale (with mask). BX: Tense; BY: Angry; BZ: Tired; CA: Unhappy; CB:Animated; CC: Confused; CD: Hurt by past actions; CE: Agitated; CF: Apathetic; CG: Mad; CH: Sad; CI: Active; CJ: To burst; CK: Irritable; CL: Dejected; CM:Energetic; CN: Out of control; CO: Hopeless; CP: Relaxed; CQ: Clumsy; CR: Rancorous; CS: Uneasy; CT:Restless; CU: Lonely; CV: Unlucky; CW: Stunned; CX: Cheerful; CY: Embittered; CZ: Exhausted; DA: Anxious; DB: Fighter; DC: Depressed; DD: Desperate; DE: Thick; DF: Rebel; DG: Helpless; DH: Without strength; DI: Disorientated; DJ: Alert ;DK: Upset; DL: Furious; DM: Efficient; DN: Full of energy; DO: Bad tempered; DP: Useless; DQ: Forgetful; DR: Unable to concentrate; DS: Tired; DT: Annoying;DU: Discouraged; DV: Resentful; DW: Nervous; DX: Carefree; DY: Terrified; DZ:Guilty; EA: Vigorous; EB: Insecure; EC: Weary

0= nothing

1= Little

2= moderately

3= quite

4= very much

Cell ED: Age (years)

Cell EE: Height (m)

Cell EF: Weight (Kg)

Cell EG-EZ: Questionnaire about the causality factors of COVID-19 pandemic” (CPFC-COVID-19). EG: “I think that restricting the movement of citizens to stop the spread of COVID-19 is unnecessary”; EH: “I think that stress influences the risk of contagion by COVID-19”; EI: “I think that if I go down the street without a mask I am not putting anyone at risk of contagion”; EJ: I think that depression due to the pandemic situation can influence the risk of contagion by COVID-19; EK: “I think that the obligation to stay at home does not prevent the spread of COVID-19”; EL: “I think that the negative psychological impact produced by COVID-19 can increase the risk of contagion”; EM: “The use of masks does not serve as a measure to avoid infections”; EN: “I think that ignorance generates a collective psychosis that induces fear that can unnecessarily maximize the risk of contagion”; E0: “Have a healthy lifestyle reduces the chances of contracting COVID-19”; EP: “I think that the population is not well informed about the political and social situation related to stopping COVID-19”; EQ: “I think that the pandemic only affects whether you maintain contact with older people or people with chronic illnesses”; ER: “I have listened to talks on the internet by experts on the subject and there is controversy about the causes of the risk of contagion”; ES: “I have read articles from scientific journals regarding COVID-19 and I do not know if the risk of contagion is high or low”; ET: “I think that the personal actions that the population is taking to try to limit the spread of COVID-19 are useless”; EU: “I am sure that I will not get infected if I go with my group of friends”; EV: “I think that the government and the media talk about too many containment measures that are not necessary to avoid contagion”; EW: “The COVID-19 pandemic has made me feel worse than I did before and I believe it has influenced my immune system, increasing my risk of contagion”; EX: “I wouldn't mind going to low-traffic rural areas without a mask since it doesn't pose any risk of contagion”; EY: “I deliberately try not to watch the news as I think information about the spread of COVID-19 may be contradictory”; EZ: “The possibility of being infected by another person without or with a mask is the same”

1= Totally disagree

2= Somewhat agree

3= Quite agree

4= Totally agree

Cell FA-FU: The Spanish version of the Basic Needs Satisfaction in General Scale (BNSG-S). FA: “I feel that I am free to decide for myself how to live my life”; FB: “I really like the people I associate with; FC: “I often don't feel very competent”; FD: “I feel pressured in my life”; FE: “People I know tell me I'm good at what I do”; FF: “I get along well with the people I usually interact with”; FG: “I close myself a lot and I don't have many social relationships”; FH: “I generally feel free to express my ideas and opinions”; FI: “I consider that the people I interact with frequently are my friends”; FJ: “Recently I have been able to learn new and interesting skills”; FK: “In my daily life I often have to do what I'm told”; FL: “The people around me in my life care about me.”; FM: “Most days I feel like I'm successful at what I do”; FN: “The people I interact with every day take my feelings into account”; FO: “In my life I don't have many opportunities to show how capable I am”; FP: “There aren't many people I have a very close relationship with”; FQ: “I feel that in my daily life most of the time I can be myself”; FR: “I feel that in my daily life most of the time I can be myself”; FS: “I often don't feel very capable”; FT: “I don't have many opportunities to decide for myself how to do things in my daily life”; FU: “People are generally pretty nice to me”

1= Not true – 7= Totally true

Cell FV-GG: The Spanish versión of The Psychological Needs Thwarting Scale (EFNP). FV: “I feel unable to make decisions regarding my life”; FW: “There are times when I feel incompetent because others have unrealistic expectations of me”; FX: “I feel rejected by those around me”; FY: “I feel pressured to behave in a certain way”; FZ: “There are times when I have been told things that make me feel incompetent”; GA: “I feel that others may have a derogatory attitude towards me”; GB: “I feel obligated to follow the decisions of others”; GC: “There are situations where I feel incapable”; GD: “I feel like other people don't like me”; GE: “I feel pressured to take on previously planned activities”; GF: “I feel incompetent because I am not given the opportunity to develop my potential”; GG: “I notice that some of the colleagues I surround myself with are envious when I am successful”

1= Totally disagree; 7= Totally agree.

Cell GH-HE: C social anxiety questionnaire (CASO N-24). GH:“Have to talk to a teacher”; GI: “That they make a joke on me in front of others”; GJ: “Participate in a school play”; GK: “Being with other girls I don't know”; GL: “Sing in public”; GM: “Go out to the board”; GN: “Play with a group of girls I know Little”; GO: “Ask a question in class”; GP: “dance in front of people”; GQ: “Telling a friend not to take my things without my permission”; GR: “Ask something from a colleague I barely know”; GS: “Make a fool of yourself in front of others”; GT: “Start a conversation with the boy I like”; GU: “let them criticize me”; GV: “Tell a colleague that I didn't like what she said about me”; GW: “Start talking to girls I don't know”; GX: “Telling a guy that I like something about him (for example, her smile, her hair, etc.)”; GY: “Telling a colleague not to bother me when I'm working”; GZ: “Losing my voice, stuttering, or having my voice shake when speaking”; HA: “Kissing the boy I like for the first time”; HB: “Let the teacher ask me in class”; HC: “Tell a colleague not to always be the center of attention”; HD: “Playing a musical instrument in public”; HE: “Ask out the boy I like”

1= not at all

2= a Little

3= quite a lot

4= a lot

Cell HF: “Do you think that these situations would have caused you greater fear, shame or nervousness before the pandemic and the obligation to wear a mask at school?”

1= not at all

2= a Little

3= quite a lot

4= a lot
