# Supplementary material for: Relationships among COVID-19 causal factors perceived by children, basic psychological needs and social anxiety
Source: PeerJ. 2025 Jan 31;13:e18828. doi: 10.7717/peerj.18828 (PMC11789665; doi:10.7717/peerj.18828)
Supplement: Supplemental Information 4 [file peerj-13-18828-s004.docx]

#import the datas

plspm <- read.table("plspm44.csv", sep=";", header=TRUE, na.strings = "")

#verify the type of variables (num)

str (plspm)

############

# construction of the coding line for the plspm model

############

# import package plspm

library("plspm")

# lines of the path matrix

DSP = c(0, 0, 0, 0, 0, 0, 0, 0, 0, 0, 0, 0, 0, 0, 0, 0)

IPP = c(0, 0, 0, 0, 0, 0, 0, 0, 0, 0, 0, 0, 0, 0, 0, 0)

ES = c(0, 0, 0, 0, 0, 0, 0, 0, 0, 0, 0, 0, 0, 0, 0, 0)

CIP = c(0, 0, 0, 0, 0, 0, 0, 0, 0, 0, 0, 0, 0, 0, 0, 0)

TENS1 = c(1, 1, 1, 1, 0, 0, 0, 0, 0, 0, 0, 0, 0, 0, 0, 0)

DEP1 = c(1, 1, 1, 1, 0, 0, 0, 0, 0, 0, 0, 0, 0, 0, 0, 0)

COL1 = c(1, 1, 1, 1, 0, 0, 0, 0, 0, 0, 0, 0, 0, 0, 0, 0)

VI1 = c(1, 1, 1, 1, 0, 0, 0, 0, 0, 0, 0, 0, 0, 0, 0, 0)

FA1 = c(1, 1, 1, 1, 0, 0, 0, 0, 0, 0, 0, 0, 0, 0, 0, 0)

CONF1 = c(1, 1, 1, 1, 0, 0, 0, 0, 0, 0, 0, 0, 0, 0, 0, 0)

TENS2 = c(1, 1, 1, 1, 0, 0, 0, 0, 0, 0, 0, 0, 0, 0, 0, 0)

COL2 = c(1, 1, 1, 1, 0, 0, 0, 0, 0, 0, 0, 0, 0, 0, 0, 0)

DEP2 = c(1, 1, 1, 1, 0, 0, 0, 0, 0, 0, 0, 0, 0, 0, 0, 0)

FA2 = c(1, 1, 1, 1, 0, 0, 0, 0, 0, 0, 0, 0, 0, 0, 0, 0)

VI2 = c(1, 1, 1, 1, 0, 0, 0, 0, 0, 0, 0, 0, 0, 0, 0, 0)

CONF2 = c(1, 1, 1, 1, 0, 0, 0, 0, 0, 0, 0, 0, 0, 0, 0, 0)

# create the path matrix (structural model = inner model) through the line of the path matrix

plspm_path = rbind(DSP, IPP, ES, CIP, TENS1, DEP1, COL1, VI1, FA1, CONF1, TENS2, COL2, DEP2, FA2, VI2, CONF2)

# add the names of the colomn within the path matrix

colnames(plspm_path) = rownames(plspm_path)

# specifi the manifest variables of all the latent variable (measurement model = outer model)

plspm_blocks = list(1:5, 6:11, 12:16, 17:20, 21:29, 30:44, 45:56, 57:64, 65:71, 72:78, 79:87, 88:100, 101:115, 116:122, 123:130, 131:137)

# create the mode vectors (A = reflective et B = formative)

plspm_modes = c("A", "A", "A", "A", "A", "A", "A", "A", "A", "A", "A", "A", "A", "A", "A", "A")

# run the partial least square path modeling analysis

plspm_pls = plspm(plspm, plspm_path, plspm_blocks, modes = plspm_modes, boot.val = TRUE, br = 100)

# outputs of the plspm results

summary(plspm_pls)

# plotting results (structural model)

plot(plspm_pls)
